# Automatic Classification of a Taxon-Rich Community Recorded in the Wild

**DOI:** 10.1371/journal.pone.0096936

**Published:** 2014-05-14

**Authors:** Ilyas Potamitis

**Affiliations:** Technological Educational Institute of Crete, Department of Music Technology and Acoustics, Crete, Greece; University of Pavia, Italy

## Abstract

There is a rich literature on automatic species identification of a specific target taxon as regards various vocalizing animals. Research usually is restricted to specific species – in most cases a single one. It is only very recently that the number of monitored species has started to increase for certain habitats involving birds. Automatic acoustic monitoring has not yet been proven to be generic enough to scale to other taxa and habitats than the ones described in the original research. Although attracting much attention, the acoustic monitoring procedure is neither well established yet nor universally adopted as a biodiversity monitoring tool. Recently, the multi-instance multi-label framework on bird vocalizations has been introduced to face the obstacle of simultaneously vocalizing birds of different species. We build on this framework to integrate novel, image-based heterogeneous features designed to capture different aspects of the spectrum. We applied our approach to a taxon-rich habitat that included 78 birds, 8 insect species and 1 amphibian. This dataset constituted the Multi-label Bird Species Classification Challenge-NIPS 2013 where the proposed approach achieved an average accuracy of 91.25% on unseen data.

## Introduction

There are many questions that scientists are called to address regarding the state of knowledge of global biodiversity. For all taxonomic groups only a percentage of 10–20% is known and logged. Even as regards known groups, their population, distribution and dynamic changes are mostly unknown to us. The urgent problem of grave importance is to be able to assess when an ecosystem reaches a degraded state to the point of irreversibility and answer such questions as ‘What is the state of the habitat in order to design policies and take conservation action to achieve sustainable use of the environment?’ or ‘How is climate change related to rates of species loss and migration?’.

Biodiversity monitoring provides the essential information on which conservation action is based. The monitoring process is typically carried out by qualified humans who observe and write down notes while taking logs of instruments that concentrate on the particular observation site. While the ability of a qualified scientist can be unparalleled to any kind of machine, there are also some limitations in human monitoring. The ability and the qualifications of the observers vary and this introduces a bias [1] in the total assessment. In addition, human expeditions are costly and can cover a limited number of sites and only work for a limited time. Moreover, the monitoring process can become more limited or even dangerous in remote, inaccessible areas. Besides, it can also be obtrusive on the species of observation.

Automatic acoustic monitoring of biodiversity is a means to provide information on species diversity with a view to the ones that are endemic, in a threatened or endangered status, or have special importance serving as indicator species. Moreover, each species is unique of its kind but can also have an additional importance due to its social, scientific, cultural or economic role.

Acoustic monitoring is limited to the part of the fauna that emits sound (birds, certain insects, certain amphibians, bats etc), which is only a subsample of the total biodiversity. However, inferring the correct taxa of this fauna and their densities can also give us an indirect cue for non-emitting sound species through their inter-dependence in the life-cycle, since animal populations are correlated.

Acoustic monitoring of biodiversity becomes, with time, a more attractive approach, as autonomous recording units (ARUs) become more affordable, can be power sufficient and can transmit their data through GSM or satellite connections from remote areas straight to the laboratory that can be located as far as in another continent. Therefore ARUs will eventually allow gathering of observations at larger spatial and time scales.

The challenge that comes along with such approach is the processing and logging of the deluge of data coming from the network of these sensors. Considering that ARUs can be operated in 24/7 modus and that several recorders can be used simultaneously, huge amounts of audio data can be gathered in relatively short periods of time. It is hardly feasible for human experts to listen to or visually inspect the complete sample of recordings. Thus semi-automatic processing of the sound files is a prerequisite for analysing the information within reasonable time limits.

The possible applications of acoustic monitoring in terrestrial environments using microphone arrays are thoroughly presented in [2]. Acoustic entropy indices [3]–[4] are also proposed as a means to assess the type of the habitat and the extent of human intervention.

There is a rich literature on automatic species identification of specific target taxon like birds [5]–[7], amphibians [8], cetaceans [9], insects [10], fish [11], bats [12] and mice [13]. All these approaches are valuable as they pave the way for automatic analysis of animal vocalisations. However, they have not yet proven generic enough to scale to taxa and habitats other than the ones described in their original publication. Moreover, the misses and false alarms of these methods will cause a very large number of cases to be further investigated manually for a system that works on a 24/7 basis. Clearly the classification scores must face the high level of false positive and false negative results in order to make acoustic monitoring practical.

The present work is an attempt to shed some light on the possibility of monitoring taxon-rich communities recorded in the wild. It is our belief, shared by others, that in order to have solid progress in this research field, researchers should depart from using private data and focus on unprocessed data as typically recorded in nature. This paper introduces our approach that took part in a taxon-rich classification challenge organized by NIPS 2013 conference for bioacoustics [14] that involves 87 species (78 birds, 8 insects and 1 amphibian) in unprocessed wild life recordings. Competition challenges are very useful as a means to put theories into practice and assess what is the state of the art, as all competing approaches are bound by a common corpus; moreover, the assessment is guaranteed not to be biased towards a private approach, as the organizers of the challenge have the responsibility of assessing the approaches and base their final ranking on data that are unknown to the contestants [15].

We have examined all reported approaches to bioacoustic challenges [15], [18], [23] to find structural parallels among the best performing approaches, gather candidate functional sub-components, integrate our novel features, and finally shape our personal strategy towards the task of classifying large taxa of animal vocalisation in unprocessed field recordings. Our research concluded into three key observations that we will thoroughly analyse in the following sections:

- The treatment of sound as picture through the spectrogram as well as the application of image-based transformations to identify local properties of the target spectral blobs can have certain advantages over frame-based audio classification approaches to the task of species identification in noisy, real environments. This work belongs to the research trend the treats audio as a picture through its spectrogram [5], [9], [16], [17].
- Multi-instance, multi-label approaches have an indisputable advantage over single- label approaches in species classification tasks [5].
- The synthesis of heterogeneous features is beneficial as it can provide complementary information that can be naturally integrated into the framework of decision trees in random forest ensembles.

This paper is organized as follows: In Section entitled ‘Methods’ we present: a) the database and its main difficulties as regards species recognition, b) the feature extraction and classifier from the perspective of image analysis of spectrograms, c) the single and multi-label approaches as well as the benefits of the latter. In Section entitled ‘Results’ we analyse the fine-tuning of the pattern recognition methods, perform experiments with real-field data and analyse the results of the current work. A discussion of the results concludes this work by presenting possible extensions and summarizing the implications of the results.

## Methods

### The Database and its Specificities

The NIPS 2013 database is composed of 1687 field-recordings each containing vocalisations of 0–6 different species. A subset of 687 recordings is offered as a training set accompanied with known annotation from bioacoustics experts and we seek the species existing in the other 1000 recordings. The hardware used to collect the data was a number of ARUs placed at different locations of the French Provence and provided by the BIOTOPE society (see http://www.biotope.fr/for details). All recordings are monophonic, sampled at 44100 Hz with variable duration from 0.25 up to 5.75 secs. We did not downsample the original recordings as there were insect species singing up to 20 kHz and night birds calling as low as at 500 Hz.

**Figure 1:**
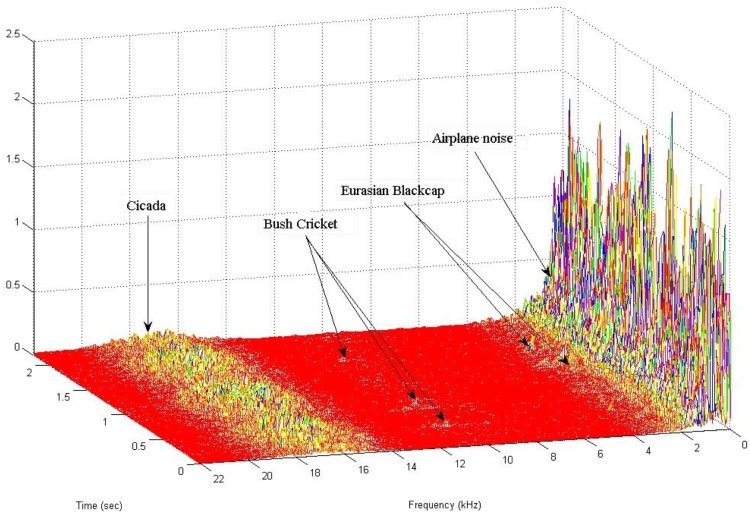
3D spectrogram of a typical recording of 3 species (trainfile115 in NIPS20134B database) demonstrating the difficulties in recognition due to different gains in target signals and noise. From 14–19 kHz a Cicada, 8–13 kHz Bush Cricket, 2.5–3.8 Eurasian Blackcap, 0–2 kHz strong airplane noise.

**Figure 2:**
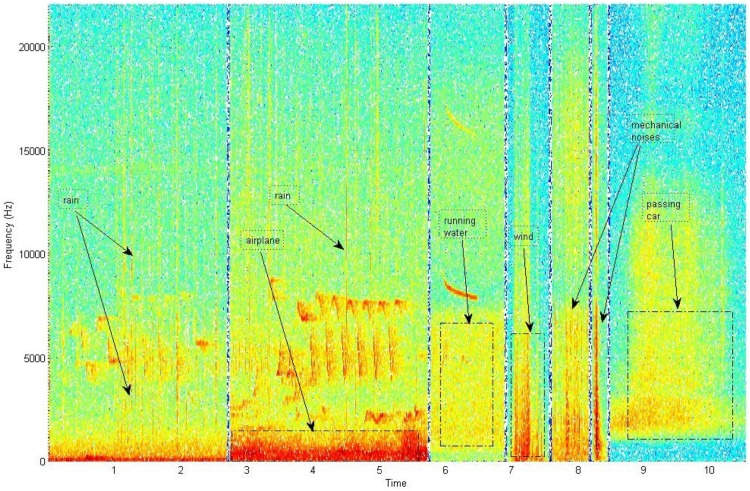
Types of anthropogenic and abiotic interfering sounds.

The training set matches the test set conditions. The list of animals can be found in [14] and contains the most encountered species in central Europe. Another unique aspect of the NIPS dataset is the distinction between calls and songs of the same species that are treated as distinct categories that the algorithms should resolve. The recordings are selected in such a way that different kinds of difficulties are encountered in almost every recording, namely:

- Different bird species can and often do overlap in the same time-frame. Vocalizations can also overlap in frequency but, do not generally overlap both in time and frequency simultaneously. This can be attributed to the sparsity of the time-frequency domain but also to the observed fact that co-existing species make use of different bandwidths in order to communicate efficiently.
- Different gains of signals due to distance: as many vocalizing animals can be at any distance from the microphone, often weak calls are picked up along with a dominating vocalization (see Fig. 1).
- Anthropogenic noises such as airplanes, sirens, footsteps, human speech in biotopes near urban territories can lead any classifier to error, because they either obscure the target signal causing a miss or become themselves classified as the target signal (false alarm).
- Abiotic sounds due to heavy wind, rain, and recording device failures can substantially reduce the quality of recordings. The sound of rain-drops and recording device failures produce sound events that are short-time with broadband spectral characteristics and can be misled with insect stridulations (see Fig. 2).

### Signal Processing & Pattern Recognition as Applied to Animal Vocalisations

#### Single- and multi-label approaches

Recognizers of animal vocalisations use prototypes of the target species extracted from examples of annotated training data. These recordings come out of large bioacoustics inventories (e.g. DORSA (http://www.dorsa.de), MacAulay Library (http://macaulaylibrary.org/index.do), Tierstimmenarchivb (http://www.tierstimmenarchiv.de), and Xeno-canto (http://www.xeno-canto.org) or from private corpora.

These inventories, at the time of their compilation did not follow a common protocol on how to log the target species. Some contain just a clip of the target species, others contain the target species in the presence of several others or in the presence of noise or they are even human narrated. The vast majority of reported research on classifying animal vocalisations follow the single label paradigm (a recording holds a single species). The reference library of a single instance recognizer should contain a large number of only the target species and nothing else, as the recognizer will learn the sound that is unrelated to the target species, which in the end will increase its false alarms. The single label approach comes with a major drawback. Not many people are qualified to recognize species efficiently by sound. In the single-instance case, the construction of a reference library is a quite laborious task as the expert must screen all recordings and apply delicate time-frequency cleaning to any possible interference. Most experts are unwilling to undertake this task for a large number of species and recordings. Again, single-instance recognizers are vulnerable to false positives, i.e. species that are not the target but sound like it and are included by mistake in the reference library. The Multi-label approach on bird recognition is in our opinion a breakthrough and was originally proposed for the task of bird recognition in [5]. In this approach the expert does not need to clean or time-stamp the recordings at all.

The expert just writes down the acoustic classes that appear in a recording e.g. European Robin, Common Chaffinch, without any particular order or time stamp. That is, he/she has to select the set of labels attributed to the recording without having to clean, remove, isolate or timestamp parts of the recording. Each recording in the NIPS 20134B database has vocalisations from a varying number of species and the training corpus is annotated to the species level. We transformed the annotation data of all recordings to a binary matrix of a dimension 687×88 (687 training files, 87 for the species and 1 for noise only recordings as seen in Table 1). Note that any all noise types as well as recording void of vocalisations can be pooled to a general noise class.

**Table 1:** Annotation sample of the training data under the multi-label framework.

#File	Noise	Long-tailed Tit	…	Turdus philomelos
001	0	1		1
002	1	0	1	1

The multi-label approach is responsible for marginalizing over the multiple labels and associate probabilities to species. This procedure accelerates the annotation of the human expert by far, as it alleviates the necessity of isolating the target signal. The expert, either by spectrographic analysis or by listening to the dubious cases, selects the appropriate tags and moves on to the next recording. Therefore, the multi-label approach relaxes by far the effort of constructing more complete reference databases.

### Pattern Matching

The automatic classification of species by machine learning techniques has a common theme. The features extracted from the unknown recordings are compared to prototypes extracted from labelled reference data in order to find possible matches. The label of the best matching reference becomes the label of the unknown recording. The prototypes can be probabilistic descriptions as in GMMs, HMMs [7], [12] or spectrographic patches serving as templates as in the general detection framework X-Bat (http://www.birds.cornell.edu/brp/software/xbat-introduction). The matching is based on calculating a distance between the target prototypes and the unknown recordings. Again the distance can be one suited against a probabilistic approach e.g. a likelihood score, a probability or a cross correlation score. The final decision on which species are to be found in an unknown recording comes after comparing the distance to a threshold.

**Figure 3:**
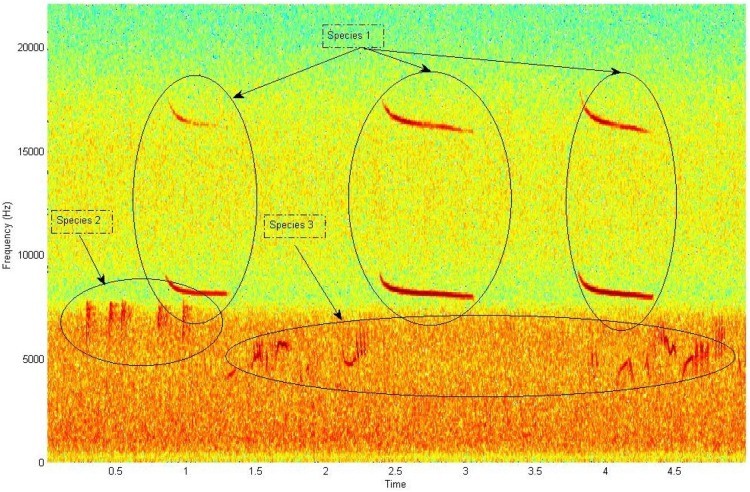
Spectrogram corresponding to a recording with 3 partially overlapping bird species (trainfile005 in NIPS20134B database). The lower part of the spectrum is coloured by the sound of running water and strong wind.

Species classification approaches based on Gaussian Mixture Models (GMMs) and Hidden Markov Models (HMMs) as typically applied to speech are related to animal vocalization classification quite well. In fact HMM’s were among the first applied classifiers following their success in speech/speaker recognition. After a thorough experimentation on private dataset of annual recordings with sophisticated versions of these tools [7] we discovered 2 major drawbacks of the HMM/GMM approach:

- Recordings in the wild can be very noisy due to their exposure to a large number of audio sources originating from all distances and directions, the number and identity of which cannot be known *a-priori*. The co-existence of the target vocalisation with other species and abiotic interferences is inefficiently treated by current approaches of audio signal enhancement and separation when the number and the nature of audio sources is unknown as when coming from an unconstrained environment. These audio sources often appear simultaneously with target vocalisations over a single time frame (see Fig. 1 and Fig. 3 for common examples). GMMs/HMMs model the features extracted from overlapping frame analysis of sound. An overlapping time-frame sound analysis will inevitably include in its spectrum some of these interferences as well as possibly and quite often the vocalisations of a number of species.
- The GMMs/HMMs species detectors derive a probability per frame (target vs. everything else -the so called ‘world model’). In the case of applying this classifier to wild-life recordings the vocalizing species will change from season to season and therefore the world-model as well. A GMM/HMM detector produces erroneous results if it is not properly updated by re-training or adapting to new species. The update procedure requires an expert in birds’ vocalisations to be available to sort out which species change and are probable to be confused with the targeted ones. This problem is not obvious when one is analysing e.g. one month of data but is prevalent in annual data analyses.

Spectrographic patches serving as templates is also one of the very first approaches employed and still is used mostly as providing a proof of concept on manually selected example recordings [25]. In unconstrained real life scenarios the variability between vocalisations of a single individual with limited repertoire not to mention variability among different individuals of the same species can be so large that templates are not capable to grasp the individuality of a target species. Moreover, spectrogram patches are vulnerable to noise and to competing species while the slightest spectral deformation can result to a large distance between the unknown vocalisation and the template.

The base classifier in our approach is a random forest under the multi-label formulation of one vs. all (the so – called binary relevance approach [5]). Random forests are an ensemble learning method for classification that is based on constructing many decision trees at training time and outputting the class that is the mode of the classes to the end of individual trees [22]. Random forests are the machine learning technique of choice here as our approach is based on deriving multiple - heterogeneous sets of features that aim to grasp different aspects of the spectrogram picture. The final dimensionality of the feature set is much larger than the size of the training set. There are few classifiers that can deal with such large dimensionalities. The class of Support Vector Machines and Extra Randomised Trees [24] that can also deal with high dimensional features were also tried out but with inferior results.

### Feature Extraction

#### The spectrogram as an image

In our work, we do not follow the framework of audio analysis but the framework of image analysis. Treating the audio scene as a picture means looking at the spectrogram as a canvas where the acoustic events appear as localised spectral blobs on a two-dimensional matrix (see Fig. 3). Once these spectral blobs are extracted, many feature extraction techniques can be applied exclusively on these spectral patches while ignoring the rest of the spectrum. In this section we describe how we extract the regions of interest (ROIs) from the spectrogram.

The recording is firstly amplitude normalized. Then morphological operations are applied on the image. These operation have as function to derive masks of spectral blobs by connecting regions of high amplitude that correspond to calls or phrases and to eliminate small regions of high amplitude that cannot belong to animal vocalizations because they are too small. Several approaches have been tried as: removing from the image a blurred version of the same image as well as removing the morphological opening of the image. First a morphological opening on an image is applied that can remove small bright spots. Opening is defined as an erosion followed by a dilation. Erosion shrinks bright regions and enlarges dark regions. Dilation has the opposite effect of erosion and enlarges bright regions and shrinks dark regions. The border segments are dropped as the lower part correspond almost always to low-pass noise spectral patches. All these different are standard approaches in image processing having as a result to remove background illumination and extract spectral blobs [21].

As an example, in the sample image (see Fig. 3), the background illumination is less bright at the bottom of the image than at the centre or top. This is due to the sound of nearby running water and wind that corrupt the low frequencies of the spectrum. After the morphological operations are applied the picture of the spectrogram is made binary in order to mark the masks of the spectral blobs.

**Figure 4:**
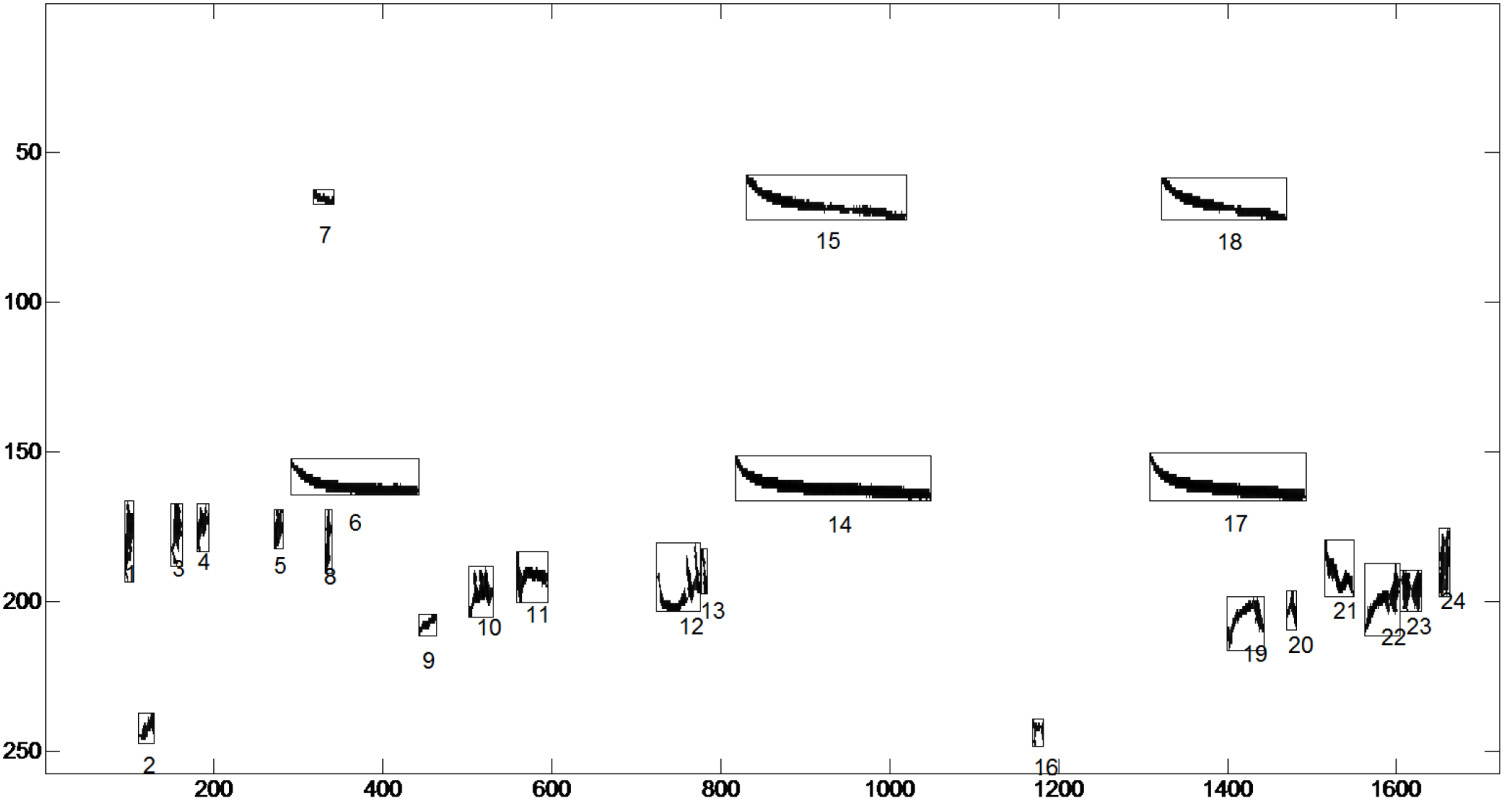
Detected spectrogram blobs of Fig. 3. Derivations and enumeration of the masks. Axis are enumerated according to their pixel index.

**Figure 5:**
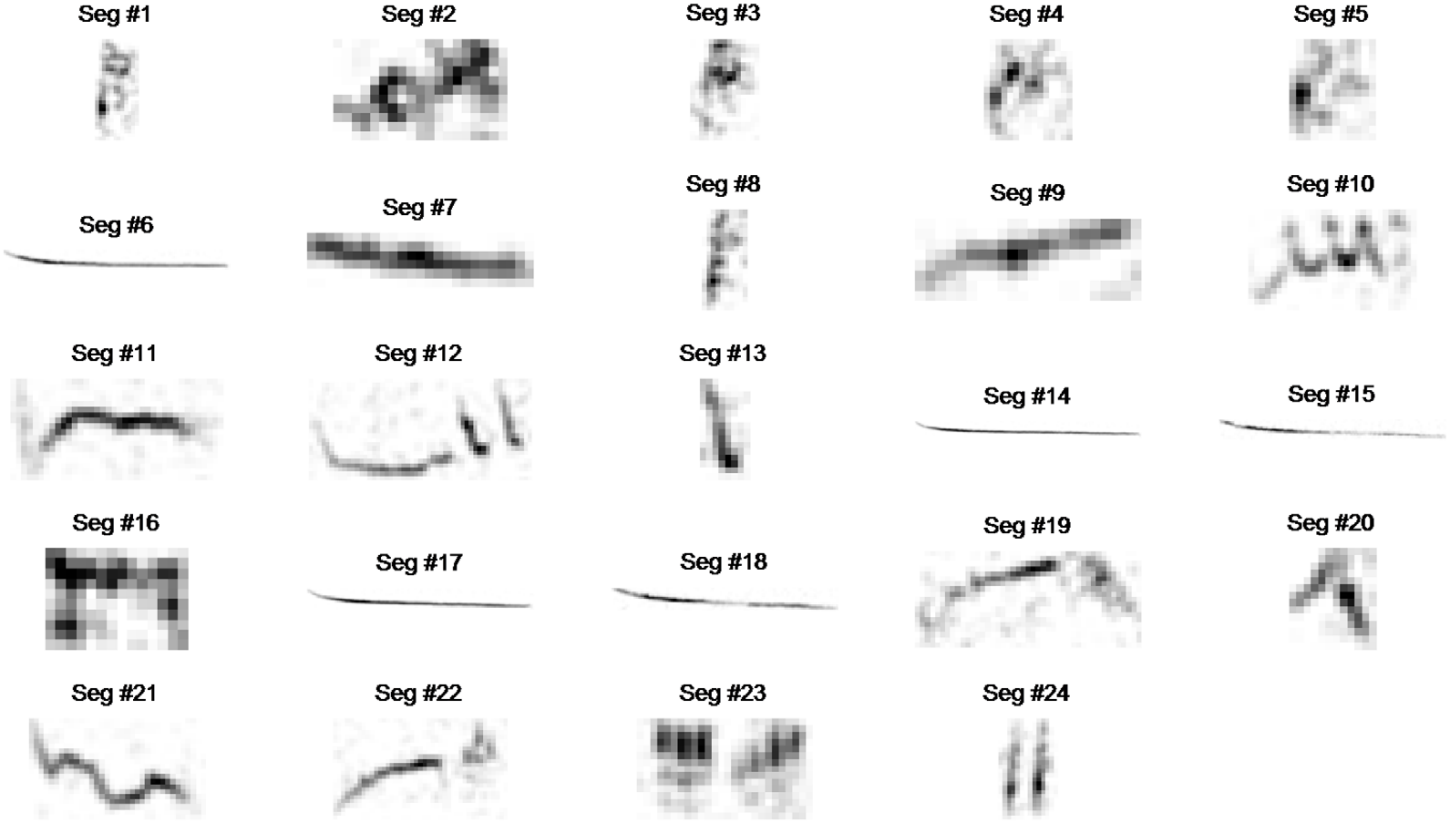
ROIs extracted after applying the masks of Fig. 4 onto the spectrogram of Fig. 3, enumerated and catalogued. The same procedure is followed for all recordings.

Binarization is realised by thresholding the image to the 90% percentile of the data (i.e. that is the highest 90% value). Subsequently we label the connected components in the 2-D binary image to derive the masks where the ROIs exist. We call ROIs the spectral patches cropped by the associated masks. Small masks (smaller than a fixed number of pixels) are discarded (see Fig. 4). The threshold for removing small masks is derived by observing short but perceptible calls from the training data and the number of pixels is set to 100. The ROIs are the patches from the original spectrogram that correspond to the pixel coordinates of the masks. All the ROIs extracted from the training set and test set are stored along with the frequency location from which they were cropped. The time information is dropped as an animal can vocalise at any time within a recording but only at the frequencies of its repertoire (see Fig. 5 for the segments extracted from the recording of Fig. 3).

Fig. 5 illustrates the ROI’s extracted from a single recording. The 16798 ROIs of all 1687 recordings are automatically extracted, enumerated and catalogued in under 12 minutes using an i7, 3.4 GHz machine. This is a distinct difference from the seminal approach of [5] where the ROIs are manually extracted, which is very time-consuming and practically impossible when the number of recordings is large. Cataloguing entails storing the spectral patches and the frequency borders in pixel coordinates from where it was extracted. Everything else but the ROIs is discarded from the data.

The benefits of extracting the ROIs are:

- The great reduction of variability between recordings. Every further analysis of our database will be done in reference to these ROIs alone while the rest of the spectrum is disregarded (compare Fig. 5 to the original Fig. 3).
- Once extracted these ROIs allow us to derive a plethora of features with gradual increase in sophistication namely: Marginal over time measurements, statistical descriptors of the shape of the ROIs and finally how the ROIs of the training set alone correlate to the spectrograms of the test set.

### Extraction of Marginal Over Time Measurements

Spectrogram reconstruction from ROIs entails that an enhanced spectrogram is extracted from the original one by imposing the ROIs of the original spectrum on an empty spectrogram (see Fig. 4). Each spectrogram is partitioned in 16 equal and non-overlapping horizontal bands. Subsequently several statistics are derived over this enhanced spectrum on a per band basis. This approach resembles filterbank analysis in audio. We do not apply mel-spacing as different birds and insects can vocalize anywhere in the spectrum. This approach allows us to make use of the band-limited character of most species and leave the marginalization process to the multi-label approach. For each band a set of basic descriptive statistics to represent the essence of each band concisely is derived from the intensities of each band, namely: Mean, standard deviation, median and kurtosis and the 90% percentile. The median is found by sorting the values and picking the middle one and is a way to summarize statistical distribution. Kurtosis (fourth central moment) is used as a measure of peaky vs. flat. In order to derive the 90% percentiles the data are ordered and the one at the highest 90% position is derived. A percentile is a statistical measure below which a given percentage of measurements in a group of measurements fall. Other descriptive statistics have been tried, such as entropy and skewness, but without presenting any statistically significant difference in the classification accuracy. Therefore, 5 descriptors for each of the 16 bands is derived resulting to a 5×16 = 80 dimensional vector per recording and a total training matrix **S_1_** ^687×80^. One should note that with this feature alone the multi-label approach achieves 86.37% average accuracy (see Results section).

### Bag of Segments, Statistics of Segments and Coding Over Multiple Codebooks

**S_1_** offers a gross description of the spectrum. The **S_1_** features set can capture quite well species vocalising in different frequency bands but it fails to capture species vocalising in the same band. Another set of features, named **S_2_** is extracted out of the examination of the morphology of each ROI. Each recording is considered as a bag of segments where the bag is the set composed of all ROIs in a recording and each ROI is an instance. For the ROIs corresponding to each recording we derive the following features:

Area of the binary mask noted as *M_area_* of the mask *M*:
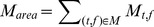
(1)where *t, f* are pixel indices belonging to the area of the mask.

Mean off the spectral patch corresponding to a ROI:
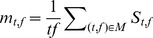
(2)where S*_t,f_* corresponds to the amplitude of the spectral chunk of the underlying binary mask *M_t,f_.*

Standard deviation of the spectral ROI:
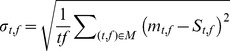
(3)


The Median, Minimum and Maximum value of the spectral patch defined by ROIs and the Maximum position of the spectral ROIs (only the frequency index retained) as well as bandwidth and duration of each ROI are set as features. Additionally, we calculate the mel-frequency cepstral coefficients (13 features) only in the frames containing the ROIs. These coefficients are derived by multiplying the amplitude of the spectrogram with a mel-scaled filterbank, then the logarithm is applied and finally the result is decorrelated using the discrete cosine transform.

However, each recording has a varying number of ROIs. In order to derive a useful description per recording this must have a fixed size so that it can be compared to the descriptions of other recordings. Therefore we follow the following steps:

- The ROIs of all training and test recordings are pooled together, whitened and clustered using a widely accepted non-supervised clustering approach namely K-means clustering [22].
- Each ROI belonging to the same recording is associated to the cluster mean from which it has the lowest distance.
- From all ROIs belonging to each recording we make a histogram of occurrences of cluster indices. This clearly has a fixed dimensionality equal to the size of the K-means partition. This procedure resembles the bag-of-words analysis used in text processing that is adapted to our bag of spectral segments case as in [5].

We have found the use of multiple code-books over the single codebook approach beneficial. A codebook with a small number of clusters will cluster using the gross-characteristic of the ROIs while gradual augmentation of the number of clusters leads to finer detail. We have been using 3 codebooks with 25, 15 and 70 clusters respectively resulting to a 687×25, 687×15, 687×70 training set which, after column stacking leads to a future set of **S_2_** ^687×110^ for the training set.

### Spectrographic Cross-correlation of ROIs

The final feature set **S_3_** is the most powerful one and has originally appeared in [19]. The key idea is to span the acoustic space of all recordings with respect to the swarm of extracted ROIs. That is, the swarm of the ROIs extracted from the training set scans the recordings and the highest normalized cross-correlation achieved from each ROI serves as a feature.

The Normalized 2D Cross-correlation of ROIs is calculated as follows:
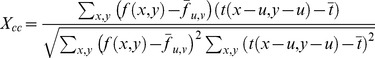
(4)
*f* is the image.


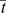
 is the mean of the template.


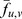
 is the mean of *f* (x, y) in the region under the template.

This feature set alone surpasses all others combined. We attribute its success to the following facts:

- Each ROI scans each spectrogram of the test set in the bandwidth limits of the ROI (enlarged±4 pixels across frequency to account for variability among individuals of the same species). Frequency constrained scanning is important since many species can have similar shapes of calls and what makes them different is their exact location in frequency.
- Lets take the case of a targeted call that must be detected in a highly degraded environment (say the Bush Cricket in Fig. 1). If this degradation does not take place exactly on the time-frequency patch of the targeted call (this is indeed the case of airplane noise in Fig. 1) this approach will locate this call even if the background noise is orders of magnitude higher. They will locate the target call because ROIs can move in a 2D search space while seeking similar patches and they can ignore everything else (including all sorts of interferences) since the path they search is constrained by the permissible frequency bounds of each ROI.
- Spectral segments are treated as wholes and do not face the problem of frame-wise processing of audio signals as GMMs/HMMs typically do.
- The set holding all ROIs derived from the training set hold different manifestations of the same call either because it is a repetition or because it comes from another individual or is a call/song from a different distance and location. Therefore, a target call/song can be tracked even if the bird has a complex repertoire (in such case the ROIs will be from different parts of the spectrum) or its call shows variations due to distance and reflections.

Since the extraction of ROIs is automatic and unsupervised spectral patches corresponding to noise will be included in the swarm of ROIs (see segments 2 and 16 in Fig. 4). In fact there will be a large number of them. The way to deal with them is to apply a selection of features during building the trees of the random forest. By monitoring the out-of-bag (oob) error and rejecting these features whose replacement does not contribute to this error we discard almost the 2/3 of the total ROIs that are really valuable for classification. The reason why most of the noise ROIs are automatically discarded is that noise patches appear spuriously and therefore do not have descriptive capability. Noise types that are not spurious such as a siren or rain are pooled in the noise class that is the 0 class in our 87 class problem and discarded during recognition as the 0 class is deleted.

One should note that detection results based on a simple cross-correlation search of a template or even multiple templates on the spectrogram return poor classification results when applied to wildlife recordings. The present approach is successful because it models the way in which the total set of ROIs fits an unknown recording and therefore integrates votes coming from all available ROIs.

A strong similarity is to be observed between our work and [20], a work developed simultaneously to but independently from ours. The difference in [20] and the proposed work is that the author presents the idea of median clipping per frequency band and time frame that removes most of the background noise. The spectrum is enhanced which results into deriving a much lower number of ROIs. Moreover he does not apply a one vs. all multi-label framework but rather isolates the ROIs that belong to each species as in [19].

## Results

### Recognition of Species

The features described in section 3 are all stacked with respect to columns forming a large training set of features **S**, where, **S** = [**S_1_**|**S_2_**|**S_3_**] and **S** ^687×16988^. Therefore an initial random forest of 80 trees is built in order to use the out of bag error to select the best performing features and reduce the dimensionality of **S** to **S** ^687×5669^. Once the features are finalized by the selection procedure the final random forest of 250 trees is constructed by training on the **S** ^687×5669^ and the associated binary multi-label matrix **Y** ^687×88^ and then applied to the test data represented by the associated matrix **T** ^1000×5669^.

The parameters of the random forest were tuned using grid-search and 10-fold cross-validation. The number of trees was set to 250 using the entropy criterion with min_samples_split = 4, min_samples_leaf = 3. The predictions submitted are averaged over 10 random forests initialized from different random seeds. The evaluation metric used was the area under the Receiver’s Operating Characteristic Curve (also known as Area Under the Curve or ‘AUC’).

Many feature combinations were tried out. We refer to the most important ones in Table 2.

**Table 2:** AUC public refers to accuracy based on the 1/3 of the test data and AUC public to the accuracy achieved using the remaining 2/3 of the data.

AUC_Public	AUC_Private	Description
0.87836	0.86368	Global Features
0.87648	0.87424	Histogram of ROIs
0.87422	0.86843	Global Features + Histogram of ROIs
0.90776	0.91310	Cross-correlation of ROIs
0.91202	**0.91689** *	Global Features + Histogram of ROIs + Cross-corr. of ROIs
0.91850	0.91752	Winning Entry
0.92251	0.91578	2^nd^ Best solution

*0.91252 achieved during contest.

**Figure 6:**
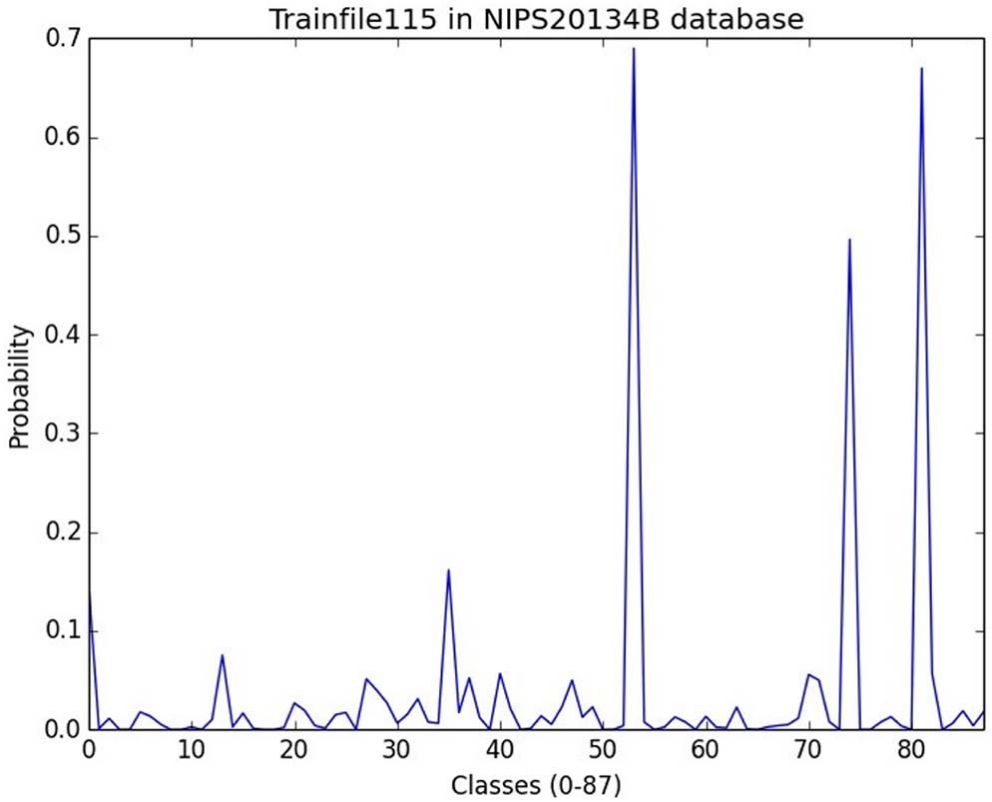
Probability output for 87 classes of the recording in Fig. 1. One can clearly discern 3 classes corresponding to the probability peaks in locations 53, 74, 81. From the file of NIPS20134B database annotations the locations 53, 74, 81 indeed correspond to Cicada, Bush Cricket and Eurasian Blackcap.

The experiments demonstrate that our approach accurately predicts a large set of species present in an unattended acoustic monitoring scenario. An example of recognizing a single audio scene is depicted in Fig. 6 where we can see a typical recognition output of the recording in Fig. 1.

### Detector of Recordings Void of Biotic Sounds

A useful by-product of building a classifier of multiple species is to make a binary classifier that decides whether there is any animal sound in a recording. The binary classifier uses the same features as for species classification (i.e. **S** ^687×16988^) but employs only the first column of the labels (i.e. if there is animal vocalization in the recording or not represented by the matrix **Y** ^687×1^). This detector can be used to derive statistics concerning activity patterns of animal vocalisations vs abiotic or anthropogenic noise sources in general. This is useful as a statistical measure of the healthiness of a habitat (which is associated to species’ vocalisation activity) and also as a means to screen out recordings that do not include animal sounds. Recordings void of any animal vocalisation are common in long-term continuous acoustical monitoring of habitats and discarding empty recordings helps to reduce the time of further processing of events as well as the storage capacity required. The test set of 1000 recordings was inspected manually and categorized into 209 recordings void of biotic sounds and 791 with at least one call per animal. The machine learning technique we used after a small set of comparisons with randomized trees and random forests was a large Gradient Boosting Classifier [22].The performance was 97.05% over a 10-fold cross-validation of the training set and 96.3% over the test set. The analytic results are depicted in Table 3.

**Table 3:** A detector of animal vocalizations vs. records containing exclusively abiotic sounds.

	precision	recall	f-score	Support
**Animal Voc.**	0.86	0.99	0.92	791
**Abiotic sounds**	0.94	0.38	0.54	209
**Avg/total**	0.88	0.86	0.84	1000

## Discussion

The probabilistic framework of random forests is very suitable for animal vocalisations classification tasks. Assuming that there are *N_tr_* annotated recordings available for training then a feature matrix **S** = *N_tr_*x*N_ft_* can be constructed where *N_ft_* represents different kinds of features stacked by column corresponding to different perspectives on how to engineer features out of spectrograms. Random forests do not need feature normalization among these heterogeneous features and can deal with dimensionality of features that can reach many thousands of dimensions. Their embedded feature selection ability during their growing effectively removes features that are not useful enough for the classification task.

As regards the computational cost deriving all features from raw recordings but the cross-correlation of ROIs (that is **S_1_** and **S_2_**) takes 13 minute on an I7 3.4 GHz PC. The training on 687 recordings and recognition of 1000 recordings takes another 10 minutes. The calculation of **S_3_** takes almost 1 day and the training and testing of the whole set about 20 minutes on the same machine. One should note that the employment of Graphical Processing Units (GPUs) is possible to reduce this computational effort to a couple of hours and we are currently working to this direction.

Another feature that in [23] was demonstrated to be highly beneficial and can potentially be integrated naturally in the statistical framework of random forests is the location of the recording device and the time-stamp of the recording. This kind of information can bias the classifier towards certain species. Location information can be inserted into ARUs during installation or through a Global Positioning System and this information can be passed automatically to the header of the sound files or to their filenames. The location of the ARUs can offer some information as regards the micro-biodiversity of the local habitat. Certain species are always found near lakes (e.g. the common kingfisher *Alcedo Atthis* or around the sea-coast etc.), while others only in rocky areas far away from lakes/coast. Species information provided by the expert can be combined with location information to provide the *a-priori* probability of a species being at this location. This probability is calculated as the frequency of a species appearing there to the total number of species tagged by the human expert during training. This probability matrix of all species serves as a feature that can be appended to the **S** matrix. The same matrix type can be calculated for the test recordings and the classifier is responsible to resolve the situation. Time information can be drawn from the internal clock of the ARU and also passed to the header of the recording or to the filename. Time information is also important as some birds show strong preference towards the time they call or sing (e.g. night-birds). The NIPS database did not include such information but; it is information often ignored while it can be obtained easily directly from the hardware of the ARUs. We deem that this information will be vital when the number of classes will reach many hundreds of species which however neither appear altogether in a certain location not they sing/call all day long.

One would then naturally ask: what are the main difficulties in recognizing taxon-rich communities in the wild using only the acoustic modality? If one could concentrate erroneous cases they would cluster due to the following reasons:

- Noise in low frequencies appears quite often and quite strong because wind is low-pass and many abiotic sounds (e.g. motors, planes etc.) are also of low-pass character and can seriously mask night-birds and other species that vocalise in very low frequencies.
- Cicadas have a noise-like spectrum that resembles the spectrum of wind when they are at large distance from the recorder and their acoustic emissions are affected by reverberation. If one applies enhancement algorithms to reduce the noise and the ROIs that will be associated with noise then one can also wipe out insect species, especially when the gain of their signal is small. If one does not apply any kind of enhancement then one will face the problem of having a large number of ROIs due to noise. Cicadas can sing for the whole duration of recordings and therefore no restriction can be applied on the size of the ROIs as in other species.
- Call repertoire of one species can be very similar at least partially to that of another to the point that even a trained ear may be in doubt (e.g., different taxa of *Parus-*tits or the notable case of the common kingfisher and the Dunnock - *Prunella modularis*). In such cases the human observer seeks other queues of information to resolve the situation (e.g. repetition patterns) that are currently not taken into account in this work.

This work assessed the potential of bioacoustic monitoring of a taxon-rich community. We have developed an approach that can monitor 78 bird species, 8 insects and 1 amphibian (a total number of 87 species under quite challenging environmental conditions). The classification accuracy was assessed by independent observers and found 91.252%.

We propose that our method is a contribution towards monitoring biodiversity at large scales through the recording of vocal fauna such as birds, insects, amphibians and mammals. We claim that the approach presented will be very robust when the training and operational data are matched and we plan to apply them at diverse environments such us underwater acoustics mainly for the recognition of cetaceans and also on bats. The classification of the immense number of recordings stemming from the deployment of a large number of ARUs will allow the following:

- Collection of relevant data to support decisions concerning the presence, absence and distribution of species.
- Data analysis in fully automated fashion that can function in degraded audio environments.
- Humans and their activities can be tracked in protected areas provided they leave an audio imprint.
